# Time perception and the experience of agency in meditation and hypnosis

**DOI:** 10.1002/pchj.276

**Published:** 2019-03-26

**Authors:** Peter Lush, Zoltan Dienes

**Affiliations:** ^1^ Sackler Centre for Consciousness Science University of Sussex Falmer UK; ^2^ School of Informatics, Chichester Building University of Sussex Falmer UK; ^3^ School of Psychology, Pevensey Building University of Sussex Falmer UK

**Keywords:** hypnosis, intentional binding, meditation, sense of agency, volition

## Abstract

Mindfulness meditation and hypnosis are related in opposing ways to awareness of intentions. The cold control theory of hypnosis proposes that hypnotic responding involves the experience of involuntariness while performing an actually intentional action. Hypnosis therefore relies upon inaccurate metacognition about intentional actions and experiences. Mindfulness meditation centrally involves awareness of intentions and is associated with improved metacognitive access to intentions. Therefore, mindfulness meditators and highly hypnotizable people may lie at opposite ends of a spectrum with regard to metacognitive access to intention‐related information. Here we review the theoretical background and evidence for differences in the metacognition of intentions in these groups, as revealed by chronometric measures of the awareness of voluntary action: the timing of an intention to move (Libet's “W” judgments) and the compressed perception of time between an intentional action and its outcome (“intentional binding”). We review these measures and critically evaluate their proposed connection to the experience of volition and sense of agency.

The experience of involuntariness is the central feature in all hypnotic responding (Weitzenhoffer, 1980). Therefore, hypnosis is characterized by changes in the sense of agency (Polito, Barnier, & Woody, 2013). The cold control theory of hypnosis posits that to respond hypnotically is to perform a voluntary action but to (intentionally) experience the action as involuntary (Barnier, Dienes, & Mitchell, 2008; Dienes, 2012; Dienes & Perner, 2007). Specifically, cold control theory predicts that the ability to respond to hypnotic suggestion reflects relatively low conscious access to information relating to intentions. Conversely, the practice of mindfulness meditation centrally involves awareness of intentions (Grossenbacher & Quaglia, 2017) and so experienced mindfulness meditators might be expected to develop improved conscious access to intentions (consistent with this suggestion, experienced meditators have been found to be less hypnotizable than nonmeditators; Dienes et al., 2016; Semmens‐Wheeler & Dienes, 2012). Empirical research into the experience of intentions over voluntary actions and the sense of agency has led to the development of temporal measures that are sensitive to intentions (Wolpe & Rowe, 2014). Here we review evidence from the application of such chronometric measures to test the theory that hypnosis and meditation are related in opposing ways to awareness of intentions. In the first section we will briefly review the measurement of time perception. In the section on Temporal measures of the sense of agency and the experience of volition, we will discuss chronometric measures related to intentions and sense of agency. Finally, in the Metacognition, hypnosis and meditation section, we will relate empirical results using these measures to theories of hypnosis and meditation.

## Temporal measures of the sense of agency and the experience of volition

### Time perception

The study of time perception involves subjective reports relating to experienced time. Time duration is typically reported by verbal estimation, duration production or reproduction, and by comparing the length of presented intervals (for a review, see Grondin, 2010). A second approach focuses on the perceived timing of specific events. The “complication experiment” method, pioneered by Wundt (1887), measures the position of a timing apparatus (initially a pendulum and later most commonly a clock) at the moment that a subjective experience of a stimulus occurs. Timing estimates generated using this method are typically compared with the objective timing of a stimulus to investigate systematic differences between objective and subjective timings. Our discussion here is limited to this second chronometric methodology.

### Awareness of intentions: Libet's clock

Libet, Gleason, Wright, & Pearl (1983) attempted to measure the time at which participants became aware of their own intention to move. Libet's participants watched an oscilloscope “clock,” which completed one full revolution every 2.56 s and reported the perceived position of the light when they experienced an urge to move. By subtracting the reported time of awareness from the actual time of movement, Libet generated a measure of the time discrepancy between subjective awareness of a “will” or urge to move and the movement itself, which he called a *W judgment.* Libet also recorded participants’ perceived time of action (or M judgments). Because the average time of onset of the readiness potential (RP) occurred before the average time of reported W judgment, Libet concluded that we become aware of our intentions after they have been initiated. Libet's proposal generated considerable controversy, with criticisms aimed at both the empirical and philosophical assumptions supporting his conclusions (Freeman, Libet, & Sutherland, 1999; see commentaries in Libet, 1987). Recently it has been argued that rather than a slow buildup of activity toward action, the RP is an artifact arising from the time locking of electroencephalography signals to movement onset, which reflects a stochastic decision process (Schurger, Mylopoulos, & Rosenthal, 2016; Schurger, Sitt, & Dehaene, 2012). Drawing on this account, Schmidt, Jo, Wittmann, and Hinterberger (2016) argue, therefore, that differences in the Libet task (e.g., such as those related to motor impulsivity; Caspar & Cleeremans, 2015) might reflect differing propensity to act on information reflected in negative deflections of slow cortical potentials.

Pacherie (2007) distinguishes between three forms of intention: future intentions (for which the goal is distal), present intentions (involving specific plans regarding the achievement of a goal in the present circumstances), and motor intentions (sensorimotor representations driving ongoing motor action in the pursuit of a goal). Although Pacherie considers W judgments to be a measure of present intentions, the timing of intentions is likely to draw on efferent information relating to motor intentions and therefore might be best considered as corresponding to Pacherie's concepts of both present and motor intentions (Gallagher, 2012). Here, the term *motor intention* will be used in a broad sense to describe the cognitive processes that may support W judgments. For example, activity in the presupplementary motor areas (preSMA) prior to movement (which, when averaged, produces the RP; Shibisaki & Hallett, 2006) is considered to at least partly support awareness of motor intentions (e.g., Lau, Rogers, Haggard, & Passingham, 2004; Libet, 1985; Libet et al., 1983).

### Sense of agency: Intentional binding

The sense of agency is the experience we have of being the initiator of our actions and controller of their outcomes (Haggard & Chambon, 2012). The experience of agency is central to human experience and, because it supports attributions of responsibility, is foundational to the formal and informal structures upon which societies depend (Haggard, 2017; Moore, 2016). Distortions of sense of agency can occur in a wide range of conditions, but are most widely recognized as a central feature of certain neurological disorders (e.g., corticobasal syndrome) and psychiatric disorders (e.g., schizophrenia; Moore & Fletcher, 2012; Rowe & Wolpe, 2015).

The sense of agency can be investigated by explicit subjective reports; for example, asking participants to respond to questions about whether or not they were responsible for a particular outcome (e.g., Ritterband‐Rosenbaum et al., 2011) or to rate how much agency they felt over a particular action (e.g., Sato & Yasuda, 2005; Wegner, Sparrow, & Winerman, 2004). Explicit reports of judgments of agency may be susceptible to demand characteristics and, given the theoretical distinction between reflective and pre‐reflective sense of agency, might influence the target of investigation (Wolpe & Rowe, 2014). Implicit measures that are sensitive to agency, but require no explicit agency‐related reflection and are therefore relatively protected against demand characteristics, are therefore commonly employed. Here we will discuss one such measure—intentional binding (Haggard, Clark, & Kalogeras, 2002).

The intentional binding effect is a compressed time interval between intentional action and outcome when an outcome (typically an auditory tone) arises from an intentional action rather than from a passive movement (Haggard et al., 2002; for reviews, see Hughes, Desantis, & Waszak, 2013; Moore & Obhi, 2012; Wolpe & Rowe, 2014). Intentional binding is closely related to causal binding, because binding occurs in passive action providing a causal relationship is believed to be present (Buehner, 2012, 2015). Indeed, when available information is closely matched across conditions, the magnitude of causal binding equals that of intentional binding (Suzuki, Lush, Seth, & Roseboom, 2019). Binding can be measured by common time perception methods; for example, duration estimate of interval between action and outcome (e.g., Engbert, Wohlschläger, & Haggard, 2008), dichotomous judgments of synchrony (e.g., Cravo, Claessens, & Baldo, 2009), or interval reproduction (Humphreys & Buehner, 2010). However, the effect was first reported using Wundt's clock method (Haggard et al., 2002). Participants report judgments of the position of a rapidly moving clock hand at the time of an occurrence of an action or of an outcome event in two conditions: a contingent condition in which the action causes the outcome, and a baseline condition in which each event occurs in isolation. These measurements are similar (and, in the case of baseline action‐timing, identical) to the M judgments employed in Libet's studies. Binding is not directly estimated but derived from judgments in different conditions. Measured in this way, intentional binding consists of opposing shifts between the perceived time of events in baseline and in contingent conditions: a shift of the outcome event toward the time of action (outcome binding) and a shift of the action towards the outcome (action binding).

### Cue combination: Mechanisms of intentional binding

Information from multiple modalities must be combined to disambiguate information streams and create stable perception of the environment (for reviews, see Ernst & Bülthoff, 2004; Seilheimer, Rosenberg, & Angelaki, 2014). One strategy for cue combination is integration by maximum‐likelihood estimation, in which the reliability of a sensory estimate is increased by combining signals from different modalities based on the relative precision (or inverse variance) of each cue (e.g., Alais & Burr, 2004; Ernst & Banks, 2002). Therefore, intentional binding may arise from the influence of the relative precision of information about action and outcome events on timing judgments (Kawabe, Roseboom, & Nishida, 2013; Wolpe, Haggard, Siebner, & Rowe, 2013). While there is existing evidence that action binding arises from a cue combination mechanism (Wolpe et al., 2013), it has been argued that outcome binding may arise when sensorimotor pre‐representation of action outcomes lowers the perceptual threshold of an action outcome (Waszak, Cardoso‐Leite, & Hughes, 2012; Wolpe & Rowe, 2014). However, outcome binding is likely to depend on temporal control rather than sensorimotor predictions of action outcomes, as binding occurs when the identity of the action outcome is unpredictable (Desantis, Hughes, & Waszak, 2012; Haering & Kiesel, 2012; Hughes et al., 2013). Furthermore, the arguments made for a dual process model are based on failures to reject the null hypothesis for differences in one of the components (e.g., Desantis, Roussel, & Waszak, 2011; Wolpe et al., 2013) and this, taken alone, does not provide evidence for the null hypothesis (Dienes, 2014). In studies where there is a reported difference in one component of binding but a failure to reject the null hypothesis for a difference in the other, it is likely that the data are merely insensitive and therefore uninformative. Therefore, there is little evidence to support a dual process model of intentional binding.

Although there has been, to our knowledge, no direct test of cue combination in outcome binding, there is indirect evidence to support the theory that both action and outcome binding arise from cue combination. For example, the disruption of activity in the preSMA by transcranial magnetic stimulation reduces outcome binding (Moore, Ruge, Wenke, Rothwell, & Haggard, 2010). The preSMA is thought to support motor intentions (for a review, see Haggard, 2008) and therefore disruption of preSMA should decrease precision of action judgments. Outcome binding is also reduced when participants are led to incorrectly believe that they did not cause an action (Desantis et al., 2011). In this case, an influence of motor intention information on the timing of an external event would be inappropriate, and this would be predicted to decrease the precision of action judgments. So, the existing empirical evidence is generally consistent with a cue combination model of both components of intentional binding. This generates simple predictions: If metacognitive access to motor intention‐related information influences the precision of action‐timing judgments, it will also influence the timing of outcome judgments, as judgments of the time at which either event occurred will be influenced by the relative precision of information relating to either event. Therefore, in cases where metacognitive access to motor signals is low and therefore precision of information about when an action occurred is relatively low, outcome binding should be relatively weak and action binding relatively strong.

## Metacognition, hypnosis, and meditation

### Metacognition of intentions and higher‐order thoughts

Metacognition can be broadly defined as cognition about cognition (Flavel, 1979). Nelson and Narens (1994) distinguish between an object level of cognitive processing and a meta‐level that monitors and controls it. The meta‐level is sometimes considered synonymous with conscious awareness (e.g., Koriat, Ma'ayan, & Nussinson, 2006), while other authors argue that metacognitive processes can be unconscious (e.g., Timmermans, Schilbach, Pasquali, & Cleeremans, 2012). According to Rosenthal's higher‐order thought (HOT) theory of consciousness (Rosenthal, 2005; for a review of HOT theories, see Carruthers, 2007), consciousness is a metacognitive process in which an unconscious first‐order cognitive state becomes conscious only when one has a HOT representing that one is in that state (Rosenthal, 2005). Such HOTs are not equivalent to introspective awareness, as a second‐order HOT will only become conscious if there is another (third‐order) HOT about it. Therefore, according to HOT theory, it is possible that intentions can occur in the absence of awareness of them. The tendency to have awareness of intentions might therefore vary both according to context and between individuals.

Subjective report of event timing can be interpreted as reflecting the availability of event timing information to HOTs. Motor action time judgments, such as Libet's M judgments or action judgments in intentional binding, require information from a range of signals, including efferent, afferent, and visual sources. For Libet's W judgments, the available information is more restricted, and may be primarily driven by early stage efferent processes (such as purportedly indexed by RPs). If binding is driven by the influence of the relative precision of auditory and action information, then motor intention‐related efferent signals will be relevant for timing judgments of both action and auditory stimuli in contingent presentations (e.g., Lush, Roseboom, et al., 2018). Therefore, Libet's W judgments and intentional binding may each reflect the availability of motor intention signals to metacognitive processes.

Note that our focus here on metacognition means we do not need to subscribe to a particular underlying mechanism of time perception. Any mechanism capable of supporting time judgments could be the target of a metacognitive process that constitutes the subjective experience of time. Differences in subjective experience of time may depend on differences either in first‐order time perception mechanisms, or just in higher‐order processes directed at them.

### Hypnosis

Hypnosis involves changes in subjective experience that arise from the delivery of imaginative suggestions within a hypnotic context (i.e., the person delivering the suggestions is designated as a “hypnotist”; Kihlstrom, 2008). Historically, much research has been directed at the question of whether or not hypnosis involves an altered state of consciousness (most commonly with regard to the concept of a “trance” state). More recently, many researchers have abandoned this question, and many researchers now agree that this theoretical distinction is not empirically useful within current conceptual and theoretical frameworks (e.g., Jensen et al., 2017; Terhune, Cleeremans, Raz, & Lynn, 2017; Woody & McConkey, 2003). However, the term *state* can be considered to describe only a probabilistic relationship between a multitude of characteristics associated with a phenomenon and to avoid attempting to draw distinct boundaries between states. With such a definition, the term *altered states of consciousness* can be meaningfully applied to hypnosis (Kihlstrom, 2018). Hypnotic responding is partly characterized by the verisimilitude or apparent reality of suggested experiences (Kihlstrom, 2008). However, the central feature common to all hypnotic responding is the experience of involuntariness over a mental or physical act (e.g., Lynn, Kirsch, & Hallquist, 2008; Weitzenhoffer, 1980).

In scientific research, trait differences in the ability to respond to hypnotic suggestion (hypnotizability) are measured by the use of standardized scales, which consist of an induction and a set of imaginative suggestions (for reviews, see Terhune & Cardeña, 2016; Woody & Barnier, 2008). Hypnotizability scores can be generated by recording dichotomous responses for each suggestion, based on behavioral indicators of a successful response (e.g., Bowers, 1993). While such “objective” scoring is commonly employed, subjective scales that allow participants to provide a quantitative measure of changes in experience may help distinguish between genuine hypnotic responding and conformity (Bowers, Laurence, & Hart, 1988; Lush, Moga, McLatchie, & Dienes, 2018). Hypnotizability can be considered a stable trait (Morgan, Johnson, & Hilgard, 1974; Piccione, Hilgard, & Zimbardo, 1989). The strongest predictor of ability to respond to an imaginative suggestion following a hypnotic induction is the ability to respond to an imaginative suggestion without an induction (Braffman & Kirsch, 1999; Kirsch & Braffman, 2001). Individual differences in hypnotizability may therefore at least partly reflect differences in a specific ability to experience involuntariness in response to imaginative suggestions.

Woody and Sadler (2008); see also Kirsch & Lynn, 1998; Lynn & Green, 2011) draw a broad distinction between sociocognitive theories and dissociation theories of hypnotic responding. Sociocognitive theories (e.g., Lynn, Rhue, & Weekes, 1990; Spanos, 1986; for a review, see Lynn et al., 2008) argue that hypnotic responding can be explained in the same terms as other social behaviors, while dissociation theories (e.g., Hilgard, 1992; Kihlstrom, 1985; for a review, see Woody & Sadler, 2008) argue for an innate mechanism that specifically supports hypnotic responding. In sociocognitive theories, hypnotic responding is goal‐directed and changes in experience occur as a direct result of contextual expectations about the hypnotic situation (e.g., that it will involve the experience of involuntariness; see Green, Page, Rasekhy, Johnson, & Bernhardt, 2006).

In dissociation theories, hypnotic responding arises from a dissociation between either cognitive control processes and behavior (dissociated control) or between cognitive control processes and experience (Woody & Sadler, 2008). The important distinction here is that in dissociated control, hypnotic involuntariness reflects a genuine lack of top‐down control, while in dissociated experience (as in sociocognitive approaches), hypnosis is goal‐directed and driven by top‐down processes. Hilgard's (1977, 1992) neo‐dissociation theory proposes that the experience of involuntariness in hypnotic responding is due to an “amnesic barrier” between the monitoring and control processes of an “executive ego” (Hilgard, 1986, p. 234), and is therefore an example of dissociated experience. Conversely, dissociated control theory (Woody & Bowers, 1994) argues that executive processes supported by the frontal lobes are weakened in hypnotic responding, so that actions are triggered without executive control by a contention scheduling system, which (according to Norman & Shallice, 1986) normally drives habitual behavior. Dissociated control approaches conflict with a large body of evidence supporting the role of top‐down cognitive processing in hypnotic responding (for a review, see Terhune et al., 2017).

Although proponents of sociocognitive approaches claim that hypnosis involves no special mechanisms over and above those used to describe other social behaviors, there is consensus that reports of hypnotically induced phenomena reflect genuine changes in experience (Lynn et al., 2008). Sociocognitive theories (e.g., Spanos, 1986) propose that changes in experience in hypnosis arise directly from, for example, expectation and motivation and appropriate strategies (e.g., directing attention, engaging in goal‐directed fantasies). A twist on this idea can be found in response set theory (Kirsch & Lynn, 1997; Lynn et al., 1990), which draws on the theory that the experience of agency is a retrospective illusion (Wegner, 2003, 2004) to argue that all behavior is unintentional. On this approach, the lack of awareness of the cognitive strategies employed to fulfill strategic goals in hypnotic responding is therefore no different to a lack of awareness of cognitive strategies in solving a mathematical puzzle (Lynn et al., 1990).

**Figure 1:**
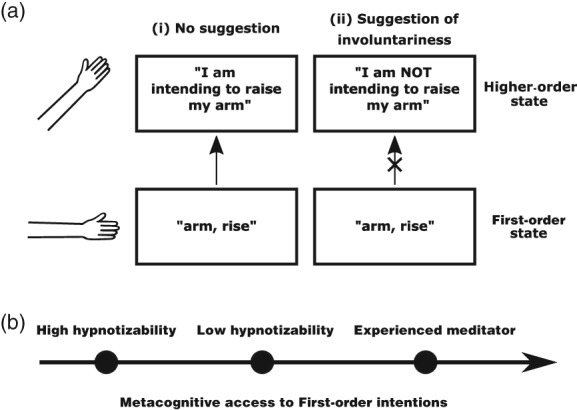
(A) The cold control theory of hypnotic responding. According to higher‐order thought (HOT) theory, a HOT of intending a motor action is based on information about unconscious first‐order intentions (i). Following a hypnotic suggestion that one's arm will move by itself (ii), first‐order intentions are preserved, but such information is avoided in forming a HOT about intention. A voluntary action is thus experienced as involuntary. (B) Trait differences in the metacognition of intentions in hypnotizability and mindfulness meditators.

The cold control theory of hypnosis (Dienes, 2012; Dienes & Perner, 2007; see also Barnier et al., 2008) provides a parsimonious unifying path through varied theoretical approaches to hypnosis. This interpretation draws on a central implication of HOT theories; intentions, as first order states, are unconscious (Rosenthal, 2008; for a review of empirical evidence for unconscious goal‐directed behavior, see Custers & Aarts, 2010). According to cold control theory, hypnotic responding is attributable to alterations in HOTs directed at first‐order intentions. For example, a successful response to hypnotic suggestion that one's arm will rise involuntarily involves an intact first‐order motor intention, but an inaccurate HOT directed at it (see Figure 1A). Therefore, hypnotic responding requires the ability to form and maintain inaccurate HOTs of intending.

Cold control theory is consistent with dissociation theories in that a particular mechanism is proposed to underlie hypnotic responding (but note that cold control theory is not only applicable to the hypnotic context and that the ability to form and maintain inaccurate HOTs of intending may support a wide variety of phenomena in which goal‐directed behavior is experienced as unintended, for example, spirit possession or channeling, automatic writing, or glossolalia; Dienes & Perner, 2007). The theory is also in agreement with sociocognitive theories that argue for a central role for expectation and context and that hypnotic responding is goal‐directed and intentional (e.g., Kirsch & Lynn, 1997; Spanos, 1986) and not with dissociated control theories. So hypnotic responding involves contextually triggered changes in the sense of agency, which may rely on an ability to form and maintain inaccurate HOTs of intending. This may reflect an ability to rely more on external cues to agency (e.g., suggestions from a hypnotist) than internal cues (e.g., motor intentions) in a hypnotic context. Dienes and Hutton (2013) report increased hypnotizability arising from disruption of the dorsolateral prefrontal cortex (a brain area that may support HOTs; Lau & Rosenthal, 2011) by repetitive transcranial magnetic stimulation (see also Coltheart et al., 2018, for a preregistered replication). Additionally, Semmens‐Wheeler, Dienes, and Duka (2013) report increased hypnotizability following administration of alcohol, which the authors argue reflects a reduction in metacognitive ability arising from alcohol‐induced disruption of the prefrontal cortex (see also evidence that alcohol reduces metacognitive awareness of mind‐wandering; Sayette, Reichle, & Schooler, 2009).

Recent work from our lab reveals differences in temporal judgments consistent with the theory that hypnotic responding is essentially metacognitive. Lush, Naish, and Dienes (2016) report the results of a Libet clock study in which groups of high, medium, and low hypnotizability (along with meditators, discussed in the following section) reported the time of an intention to move. High hypnotizables reported the latest W times, with average time in this group occurring after the movement had occurred, and low hypnotizables the earliest times.

These results are consistent with the cold control theory of hypnosis: To respond to a hypnotic suggestion is to act voluntarily whilst forming and maintaining an inaccurate HOT about that intention (Dienes, 2012). Such inaccurate metacognition requires that information related to the intention be given low weighting in the generation of a HOT of intending. Therefore, reports of delayed experience of motor intentions in high hypnotizables may reflect the relative inaccessibility of motor‐intention‐related information to higher cognitive processes. A recent study provides support for this theory outside of temporal judgment tasks; in a metacognition of agency task (Metcalfe & Greene, 2007), high hypnotizables are less vulnerable than low hypnotizables to distortions in their sense of agency brought about by disruption of control (Terhune & Hedman, 2017).

There is evidence that M judgments influence the timing of W judgments (so that W judgments are shifted earlier in time when M judgments are taken rather than not), and it has been suggested that W judgments taken in the presence of M judgments may therefore partially reflect inferences about the timing of intention relative to the time of action rather than metacognitive access to information about intentions (W judgments are earlier when participants have experience of reporting M judgments; Dominik et al., 2017). Our results contrasting meditators with hypnotizable groups were obtained without M judgments being taken (Study 1 of Lush, Naish, & Dienes, 2016). When M judgments were taken (Study 3 of Lush, Naish, & Dienes, 2016, which did not involve meditators), the results were consistent with those of Dominik et al. (and replicated the correlation between hypnotizability and W judgments).

Other evidence consistent with the predictions of cold control theory has been found in intentional binding studies. The cue combination theory of temporal binding provides a simple explanation for why binding is sensitive to intentions. The cue combination theory thus links intentional binding to cold control, showing how chronometry is relevant to cold control theory. According to cue combination theory, a difference in the relative precision of action judgments necessarily generates different action and outcome binding shifts. In intentional action, motor‐intention‐related information is available to support judgments of the time of action. In passive action, this information is not available. Intentional action therefore generates more precise judgments of action time than passive action, simply because more information about the time the action will occur is available. Thus, a theory of intentional binding—cue combination—allows precise predictions of binding to predicted differences in the availability of information about motor intentions. In a binding task conducted by groups of high and low hypnotizability in which no hypnotic induction or suggestions were preformed, low hypnotizables reported weaker action binding and more precise judgments of action timing than high hypnotizables (Lush, Moga, et al., 2018; Lush, Roseboom, et al., 2018). These results therefore support a cue combination model of binding, in which more precise information about action timing available for timing judgments should result in more influence of the action event than the outcome event in judging the time of action and therefore weaker action binding. These differences in trait hypnotizability may therefore be related to trait differences in metacognition of intentions.

It has also been demonstrated that a posthypnotic suggestion (PHS) of involuntariness over actions leads to changes in the perception of time. Haggard, Cartledge, Dafydd, and Oakley (2004) tested the effect of a PHS of involuntariness on M judgments. When participants explicitly reported experiencing involuntariness over action, judgments of the time at which an action occurred were later than when the action was performed without a suggestion of involuntariness. Lush et al. (2017) recorded explicit reports of voluntariness following a PHS of involuntariness in high hypnotizables performing an intentional binding task. When compared with voluntary action, the backward shift of outcome timing judgments towards the time of the action (outcome binding) was reduced in highly hypnotizable participants who reported a PHS‐induced experience of involuntariness over their action whilst performing the task. Importantly, outcome binding was not reduced in medium hypnotizable participants, who did not report a PHS‐induced experience of involuntariness over their actions. As intentional binding is sensitive to agency (for a review, see Moore & Obhi, 2012), this reduction in binding suggests intention‐related information is reduced in judgments of action timing during an experience of hypnotic involuntariness. This result is also consistent with a cue combination model of intentional binding, as the reduction of outcome binding in highs which accompanied reports of the experience of involuntariness over intentional action was accompanied by an increase in the variability of action judgments. Just as relatively high precision of action judgments should be reflected in relatively weak action‐timing judgments, relatively low precision of action timing should result in weaker outcome binding (as the influence of the action event over the judged time of an outcome will reduce). An increase in the variability of action judgments is suggestive of a decrease in the availability of motor‐intention‐related information for timing judgments and an intention being conscious may increase its availability to other cognitive processes (e.g., Cleeremans & Jimenez, 2002). Therefore these results can be taken as consistent with the cold control theory of hypnosis; the experience of involuntariness over a voluntary action in hypnotic responding depends upon the avoidance of intention‐related information in generating a HOT about intention, and this is reflected in relatively low precision of action‐timing judgments. Therefore, in addition to differences related to trait hypnotizability, there is also evidence consistent with changes in metacognition of intentions for a hypnosis‐related “state.”

### Mindfulness meditation

Mindfulness (a 19th century translation of the Pali word *sati*; Bodhi, 2011) is an important concept in Buddhist meditation practice, which has come to be influential in the West through its adoption in psychotherapeutic techniques, perhaps most famously in Jon Kabat‐Zinn's Mindfulness‐Based Stress Reduction Program (Kabat‐Zinn, 2011). Mindfulness meditation can be said to induce an altered state of consciousness, in a weak sense, by alterations in the focus of attention, for example toward bodily states (Manuello, Vercelli, Nani, Costa, & Cauda, 2016; Wittmann, 2015).

In Buddhist sources, there is no single definition of *mindfulness*, as the concept has developed through a wide variety of scholastic traditions (Dreyfus, 2011; Gethin, 2011). The varied definitions within traditions are often obscure (e.g., “not wobbling” or “not drifting”; Dreyfus, 2011) or established in metaphor (e.g., as a guard watching the doors of a house, Gethin, 2011). Kabat‐Zinn (2003) defines *mindfulness* as “the awareness that emerges through paying attention on purpose, in the present moment, and non‐judgmentally to the unfolding of experience moment by moment” (p. 145). This emphasis on present moment awareness and a non‐judgmental attitude toward thoughts is a common feature of Western definitions of mindfulness (e.g., Bishop et al., 2004; Kristeller, 2007). However, such an approach may mischaracterize the Buddhist concept of mindfulness, which fundamentally involves remembrance, and also making judgments about particular mental states in progressing toward a particular ethical goal (Bodhi, 2011; Dreyfus, 2011; Gethin, 2011; Kuan, 2012). Therefore, an attitude of non‐attachment or acceptance in mindfulness is perhaps better communicated by the term *equanimity*, which Desbordes et al. (2015) define as “an even‐minded mental state or dispositional tendency toward all experiences or objects, regardless of their affective valence (pleasant, unpleasant, or neutral) or source” (p. 357). This concept should be considered distinct from indifference, which, while apparently similar, can be considered as oppositional to equanimity (Bodhi, 2000); thus an attitude of curiosity is sometimes used to characterize mindfulness (compare the Pali metaphor of mindfulness as a surgeon's probe to gather information, Analayo, 2003, p. 53).

Mindfulness practice is derived from the central teaching of the Buddha on mindfulness, the *Satipatthana Sutta.* This work consists of a series of discourses (purportedly in the words of the Buddha) that present a number of meditation practices to develop mindfulness within four domains (Analayo, 2003). While the first of these domains relates mindfulness to awareness of the body, the remainder all involve awareness of mental states (Dienes et al., 2016). Therefore, the metacognitive monitoring and control of cognitive processes is centrally involved in mindfulness practice (e.g., in monitoring and redirecting attention; Bishop et al., 2004; Brefczynski‐Lewis, Lutz, Schaefer, Levinson, & Davidson, 2007).

Lutz, Slagter, Dunne, and Davidson (2008) identify two styles of meditation within an attentional family of mindfulness meditation practices common to multiple Buddhist traditions, including Zen, Vipissana, and Tibetan Buddhism. Examples of focused attention practices include *samatha* meditation within the Theravadan tradition, which has the aim of developing concentration (*samadhi*; Kuan, 2012). Focused attention meditation involves maintaining attentional focus on a single object, for example, one's own breath. Such focused attention is distinct from that common every day (for example when absorbed in an activity) as it requires the metacognitive monitoring of mental states (or “meta‐awareness,” Dahl, Lutz, & Davidson, 2015, p. 516) to prevent attention drifting from the object. Note that, contrary to secular definitions of mindfulness as nonjudgmental, this process requires assessing whether a particular mental state is consistent with intentions (Dreyfus, 2011; Gethin, 2011).

In contrast, in open‐monitoring meditation, there is no preselected object of attention. Rather, the “attentional scope is expanded to incorporate the flow of perceptions, thoughts, emotional content and/or subjective awareness” (Dahl et al., 2015, p. 516). Open monitoring practices are therefore metacognitive. Open monitoring techniques are especially related to the Zen (Chan) and Tibetan Dzogchen traditions; Theravadan insight (vipissana) meditations combine qualities of both some task focus and some degree of open monitoring. When meditation includes insight, attention expands to consider properties of mental states, such as their transience or felt ownership, relevant to the Buddhist analysis of flourishing. Novice meditators are often introduced to focused attention techniques before open monitoring, as metacognitive skills developed by focused attention meditation may aid open monitoring (Lutz et al., 2008).

Theoretical approaches that propose a key role for metacognition in mindfulness meditation may also be supported by the Buddhist literature. For example, a contemporary Buddhist scholar, Kuan (2012) finds support for interpretations of *samatha* and *vipissana* meditation as processes of metacognitive monitoring and control in the Theravadan Pali canon:

Some psychologists suggest that mindfulness corresponds to metacognition. My study shows that this correspondence can be corroborated by Buddhist literature since *sati* ‘mindfulness’ consists in steering *saññā* ‘cognition’ in such a way that one's cognition is rendered wholesome in a Buddhist sense. While mindfulness and concentration both involve attention (*manasikāra*), mindfulness in particular plays a pivotal role in regulating attention. In the case of *vipassanā* (insight) meditation, attention is regulated by mindfulness in such a way that it is not focused on a single object, but is directed to monitor the ever‐changing experiences from moment to moment in a way conformable to Buddhist doctrine, so that the practitioner attains ‘metacognitive insight’ whereby he recognizes the nature of all things as impermanent, unsatisfactory and not‐Self. In the case of *samatha* (serenity) meditation, in order to attain the state of ‘concentration,’ one has to concentrate one's attention on a single object. Mindfulness picks an object as the focus of ‘selective attention,’ that is *ekagga* ‘one‐pointedness’ in Buddhist terminology, and monitors whether attention is focused on the chosen object to ensure that the state of concentration is maintained. (p. 55)

So, there is agreement between secular and Buddhist theorists that mindfulness is a form of metacognition. While metacognition of intentions is part of the fourth application of mindfulness described in the *Satipathana Sutta* (Analayo, 2003), it is not generally presented as being of particular significance to mindfulness meditation. However, arguably metacognition of intentions is central to both focused attention and open monitoring practice. In focused attention meditation, one must sustain an intention to maintain concentration on a particular object, during which other intentions may arise, and these must be monitored and controlled in order to sustain attention. Repetti (2010) argues, therefore, that metacognition of intentions is at the core of mindfulness meditation practice, and that it develops awareness of intentions:

Meditation cultivates an increasing awareness of pre‐conscious, impersonal cognitive/volitional forces that fuel distractions, engage and direct attention, and trigger actions, and it simultaneously cultivates volitional detachment and liberation‐oriented volitions and metavolitions. As the practitioner becomes more aware of behavioral triggers, she becomes more able to refrain from acting on them. Thus, Meditation is a form of metamental training that increases volitional self‐regulation (autonomy). (p.177)

Grossenbacher and Quaglia (2017) present a parsimonious model of mindfulness meditation that places a central emphasis on metacognition of intentions. The Contemplative Cognition Framework identifies three constructs as being central to mindfulness and meditation: intended attention, attention to intention, and awareness of transient information (or present moment awareness). Here, *attention* is defined as a process that modulates the efficiency of other ongoing processes and *intention* is defined as a process of motivation that specifies a goal and makes further processing to achieve that goal more likely. *Awareness* entails conscious experience and makes cognitive representations available to other processes (e.g., Baars, 1997; Cleeremans & Jimenez, 2002). These three distinct attention‐related processes together constitute the cognitive processes that characterize mindfulness meditation. Grossenbacher and Quaglia distinguish between intentions to attend and attention to intentions, and argue that it is the interplay of these in relation to attention to transient information (in the present moment) that constitutes mindfulness meditation. Mindfulness meditation therefore involves intentions to attend in the present moment; focused attention involves an intention to pay present moment attention to a particular object (and the intention to notice when attention drifts from this object; Latham, 2016), while in open monitoring the intention is to pay attention to any mental states that happen to arise. Successfully maintaining an intention to attend in the present moment requires the metacognitive monitoring and modulation of intentions, of both the intention to attend and of any conflicting intentions that may arise.

Latham (2016) relates OM and FA meditation to HOT theories, drawing on a simple distinction between first‐order states (which are not about other mental states) and higher‐order states (which are about other mental states). On this interpretation, the intention to pay focused attention to an object (as in FA practices) is an intention to maintain a first‐order mental state, which is likely to also involve an intention to notice whenever attention shifts from the object. Fulfilling such an intention requires a HOT about the contents of the first‐order state. Open monitoring practices, on the other hand, can involve the monitoring of both first‐ and second‐order mental states by higher‐order states (depending on which mental states arise). However, OM may still involve HOTs of first‐order intentions, as such mental states may be amongst those arising during monitoring. Long‐term meditation practice may develop enhanced phenomenology of HOTs (just as experienced artists or musicians are capable of more detailed perceptions relating to their area of expertise), which in turn may improve metacognitive monitoring (Latham, 2016).

So, Buddhist meditation fundamentally involves practicing metacognition of first‐order intentions, and therefore may develop finer‐grained HOTs of intending. The centrality of awareness of intentions to Buddhist practice has been related to the experimental tradition pertaining to awareness of intentions in psychological science. For example, Dreyfus (2011) argues that mindfulness practitioners “should be able to distinguish more carefully their own intentions and the degree to which those precede their actions or fail to do so” (p. 53) and Repetti (2010) says that “meditators’ scores on the temporal disparity between neural volitions and mental volitions will be significantly less than those of non‐meditators” (p. 207). Consistent with these suggestions, there is evidence that Buddhist meditators may have improved access to negative deflections of slow cortical potentials which, when averaged, produce the RP (Jo, Wittmann, Hinterberger, & Schmidt, 2014; see also Jo, Hinterberger, Wittmann, & Schmidt, 2015; Jo, Wittmann, Borghardt, Hinterberger, & Schmidt, 2014). Furthermore, there is evidence that meditators are less hypnotizable than nonmeditators, perhaps because they have finer‐grained concepts of first‐order intentions (Dienes et al., 2016; Semmens‐Wheeler, 2012; Semmens‐Wheeler & Dienes, 2012).

As in the case of trait hypnotizability, these predictions are supported by the results of studies in which meditators report temporal judgments. In a Libet task (and in contrast with high hypnotizables) experienced mindfulness meditators report earlier awareness of an intention to move than nonmeditators, which may be attributable to the relative accessibility of motor intention‐related information (Lush, Naish, & Dienes, 2016). Mindfulness meditators also showed stronger outcome binding than age‐matched controls in an intentional binding task (Lush, Parkinson, & Dienes, 2016). While reports of illusory time perception by experienced meditators may at first appear counterintuitive, the cue combination theory of intentional binding again allows us to link cold control theory to chronometry through a proposed relationship between the availability of motor intentions and the precision of action time judgments. In cue combination models of intentional binding, the magnitude of outcome binding should be positively related to the precision of information about the timing of the action (as more precise information about an action results in a greater influence of that information over the judged time of an outcome). Therefore, improved metacognition of intentions arising from mindfulness meditation practice may drive increased outcome binding because information about the timing of action arising from efferent signals is more precise in meditators than in nonmeditators. In this way, the less veridical time perception reported by meditators in an intentional binding task may be directly linked to improvements in the availability of motor‐intention‐related information arising from meditation practice. Mindfulness meditators and highly hypnotizable people may therefore lie at different ends of a spectrum of metacognition of intentions (see Figure 1B). We are currently testing the link between mindfulness training and metacognition of intentions by testing the hypnotizability of nonmeditators before and after a period of mindfulness training. If hypnotic responding requires relatively low access to motor‐intention‐related information in the generation of HOTs of intending, training in awareness of intentions should reduce hypnotizability. Consistent with this proposal, mindfulness meditators have been reported to be less hypnotizable than nonmeditators (Semmens‐Wheeler & Dienes, 2012; Semmens‐Wheeler et al., 2013).

Note that there may be more than one route to successfully responding to an imaginative suggestion within a hypnotic context. Highly hypnotizable people may be divided into subtypes, broadly distinguished by whether they achieve responses through a dissociative mechanism or by cognitive strategies (Barber, 1999; Terhune, Cardeña, & Lindgren, 2011). The theory presented here would apply only to a dissociative subtype on this distinction. It might be possible, therefore, for experienced meditators to successfully respond to hypnotic suggestion if the response is achieved through cognitive strategies rather than dissociation of HOTs from first‐order intentions.

Although metacognition of intentions is, arguably, a central aspect of mindfulness (Grossenbacher & Quaglia, 2017), Buddhist practice involves mindfulness of a wide variety of perceptions (e.g., Dreyfus, 2011; Kuan, 2012) and there is no reason to expect mindfulness‐related differences in the formation and maintenance of HOTs to be limited to those directed at intentions. We might therefore expect meditators to also have improved metacognition other than of intentions (e.g., see Fleming & Lau, [Link]). Applying other metacognitive measures to hypnotizable groups could also inform theories of hypnosis. Cold control theory does not specifically predict domain‐general changes in metacognition and evidence for a relationship between domain‐general metacognition and hypnotizability would require going beyond the theory.

## Conclusion

We have presented evidence in support of the claim that hypnosis and mindfulness meditation are related to metacognition of intentions in opposing ways; the practice of mindfulness meditation may develop metacognition of intentions, while trait differences in the ability to respond to hypnotic suggestions may reflect differences in the availability of first‐order intentions to HOTs. We argue that it is trait differences in metacognition of intentions that drive differences in time perception in meditation and hypnotizability.

### Disclosure of conflict of interests

The authors declare that there are no conflicts of interest.
